# Corrosion Degradation Mechanism of Cr-Coated Zr-4 Alloy under Simulated Nuclear Conditions for Accident-Tolerant Fuel

**DOI:** 10.3390/ma17061240

**Published:** 2024-03-07

**Authors:** Yanfeng Wang, Juanjuan Geng, Yun Wang, Shaopeng Wang, Changwei Zhang

**Affiliations:** Northwest Institute for Nonferrous Metal Research, Xi’an 710016, China; gjjnin@163.com (J.G.); ywang1321@sina.com (Y.W.); pengfly239@163.com (S.W.); 15114849858@163.com (C.Z.)

**Keywords:** Cr coating, arc ion plating, isothermal oxidation, accident-tolerant fuel

## Abstract

Cr coatings with a thickness of about 19 μm were synthesized on Zr-4 cladding using plasma-enhanced arc ion plating. A Zr-Cr micro-diffusion layer was formed via Cr ion cleaning before deposition to enhance the interface bonding strength. Cr coatings exhibit an obvious columnar crystal structure with an average grain size of 1.26 μm using SEM (scanning electron microscopy) and EBSD (electron backscatter diffraction) with a small amount of nanoscale pores on the surface. A long-term aqueous test at 420 ± 3 °C, 10.3 ± 0.7 MPa and isothermal oxidation tests at 900~1300 °C in air were conducted to evaluate the Cr-coated Zr-4 cladding. All the results showed that the Cr coatings had a significant protective effect to the Zr-4 alloy. However, the corrosion deterioration mechanism is different. A gradual thinning of the Cr coating was observed in a long-term aqueous test, but a cyclic corrosion mechanism of void initiation–propagation–cracking at the oxide film interface is the main corrosion characteristic of the Cr coating in isothermal oxidation. Different corrosion models are constructed to explain the corrosion mechanism.

## 1. Introduction

**Figure 1:**
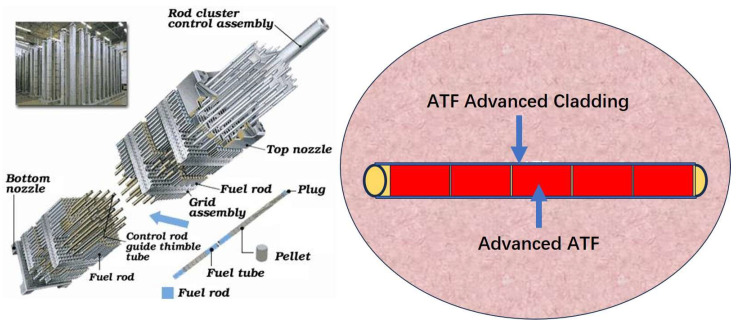
Illustration of nuclear fuel assembly and advanced ATF mode.

As there is a growing demand for environmentally sustainable electricity generation, nuclear power generation has attracted considerable attention for its low carbon emissions and safe waste disposal. However, the Fukyshima Dai-ichi nuclear accident in 2011 has cooled the development of nuclear power due to the instability of the current UO_2_ pellet-Zircaloy system in high-temperature water. After that, the accident-tolerant fuel (ATF) program, aimed to enhance the stability and safety of nuclear reactors under normal or off-normal conditions [1,2,3,4], has become one of the key focuses of the nuclear industry. As shown in Figure 1, UO_2_ pellets are placed inside a zirconium alloy cladding tube to form a fuel rod, and numerous fuel rods are combined to form a fuel assembly. For the ATF, there are currently two ways: one is developing advanced fuel pellets and improving the reaction controllability, and the other is utilizing advanced cladding and enhancing its service stability in high-temperature water. However, developing advanced fuel pellets and fuel cladding requires extensive research and experimentation, which is a long-term process for ATF.

In the short-term-plan of the ATF program, coating technology is considered to be a promising approach owing to its low R&D (research and development) costs, short R&D times and minimal impact on existing fuel systems. Candidate coatings for ATF are required to exhibit high corrosion resistance in light water reactor (LWR) coolant environments, good structural stability under neutron irradiation, and excellent oxidation resistance against high-temperature steam [5]. Specifically, under the off-normal condition of a nuclear reactor, the reaction between Zirconium and high-temperature water can be completely avoided by that protective coating, thereby inhibiting the occurrence of a serious hydrogen explosion accident [6,7,8]. Various types of coatings have been studied, such as metal materials (FeCrAl [9,10] and Cr [8,11,12]), MAX phase materials (Ti_3_AlC_2_, Cr_2_AlC, Ti_3_SiC_2_) [13,14] and ceramic coatings (SiC [15,16], CrN [5]).

The FeCrAl coating performed well in high-temperature conditions due to the growth of a dense alumina scale. However, the serious inter diffusion between Fe and Zr at temperatures over 1000 °C would prematurely damage the coating by inducing the low melting point eutectic phase at the interface [17]. The MAX phase (M represents transition metal elements; A represents the main family element; X represents carbon or nitrogen) coatings exhibited good corrosion resistance and radiation damage resistance, whereas the interdiffusion is still a challenge due to the high activity of A-site atoms. The nitride phase will release nitrogen in high-temperature water [18] and also the intermediate compound, such as ZrAl which can be easily formed by diffusion [19], leading to the deterioration of the coating. In addition, the alumina- and silica-forming MAX coatings may not provide effective protection in LWR conditions, since Al_2_O_3_ and SiO_2_ can be decomposed to AlOOH and H_4_SiO_4_ in high-temperature water [20,21,22]. Ceramic coatings are susceptible to cracking and spalling under severe thermal shock due to their inherent brittleness, resulting in premature failure.

Among the numerous coatings for ATF, the Cr coating is considered to be the most prospective material due to its excellent oxidation resistance, low thermal neutron absorption cross-section, high neutron irradiation resistance, and favorable thermo-mechanical properties [23,24,25,26]. Various techniques have been used to fabricate Cr coatings. For instance, over-100-μm thick Cr coatings were deposited using a laser beam or spraying techniques, which provided good protection for Zr claddings in high-temperature steam [27,28,29,30,31,32]. Unfortunately, the protection of these Cr coatings would quickly deteriorate due to the rapid penetration of corrosive media through the primary defects of pores and cracks. Besides, the Cr coatings produced by these techniques typically possess rough surfaces, and require an additional surface treatment process for further application. Hence, it is essential to develop a suitable technique to produce more compact and smooth Cr coatings.

Physical vapor deposition (PVD) technology has a great advantage in depositing high-quality Cr coatings [12,25,26,32]. All the reports show that the PVD-Cr-coated claddings have exhibited excellent corrosion resistance in high steam at 1200 °C with the forming of a dense Cr_2_O_3_ on the surface, while the uncoated inner surface was extremely oxidized. Since the Cr coating is totally consumed in high-temperature steam, X. Han et al. have proposed an oxidation mechanism based on the diffusion and redox reaction between the Zr and Cr_2_O_3_ layers, which is more complicated than generally assumed [33]. The performance and degradation mechanism of Cr coatings under extreme accident conditions have been widely investigated. However, far too little attention has been paid to the durability of Cr coatings in normal LWR or even harsher environments, which is a critical consideration for engineering applications.

Hence, in this study, Cr coatings were deposited on the Zr-4 samples and claddings via a plasma-enhanced arc ion plating (AIP). A relatively rigorous long-term aqueous test at 420 ± 3 °C, 10.3 ± 0.7 MPa for 100 days in deionized water was designed to evaluate the durability of the Cr coating under the off-normal LWR service condition rapidly. And isothermal oxidation tests at 900~1300 °C in air were also carried out to study the protection of the Cr coating at a supercritical design temperature. Furthermore, the degradation of Cr coatings under different conditions was discussed.

## 2. Materials and Methods

The Zr-4 alloy (basic chemical composition of this alloy was listed in Table 1) parallel specimens and claddings (Produced by Western New Zirconium Nuclear Materials Co., Ltd., Xi’an, China) were polished to a surface roughness below Ra 0.2 with Al_2_O_3_ abrasive paper. Then, the samples were immersed into a mixed acid solution of 5% HF, 45% HNO_3_ and 50% H_2_O, lasting about 30 s, cleaned by alcohol and dried in warm air. Cr coatings were deposited on these Zr-4 samples using a plasma-enhanced arc ion plating (AIP) system (TSU-1000, Beijing Tecno Technology Co., Ltd., Beijing, China). The ionization rate of Ar and Cr can be enhanced by the assisted plasma, which is beneficial to improve the densification of Cr coatings. Prior to deposition, the system was evacuated to a vacuum pressure of 5 × 10^−4^ Pa. A two-step ion cleaning process was used to ensure the cleanliness of the samples. Firstly, the glow Ar+ cleaning process under a bias potential of 1100 V was employed to remove the slight contaminants and static charges. Secondly, the metal Cr ion cleaning driven by high-purity Cr targets at a pulse-bias potential of 800 V was executed. This Cr ion cleaning could activate the surface of Zr-4 substrate and form a certain zone of Cr ion implantation with compressive stress, which is beneficial for the interfacial bonding strength. After that, the Cr coatings were deposited by setting the parameters as follows: a pulse-bias potential of 60 V, gas pressure of 0.8 Pa, Cr target current of 80 A, deposition temperature of about 220 °C. By adjusting the deposition time, Cr coatings with thicknesses of about 20 μm were deposited on the Zr-4 samples.

**Figure 2:**
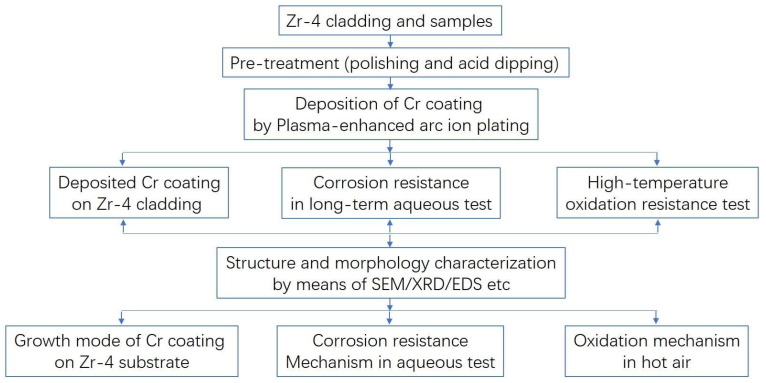
Illustration of the sample preparation procedure and plan of experiments.

Under accident conditions, the temperature inside the reactor can quickly reach a critical value of 1200 °C or even higher. So, it is necessary to characterize the ability of the Cr coating under extreme working conditions. In this article, two kinds of test are carried out: the isothermal oxidation test and long-term aqueous test. In terms of isothermal oxidation test, the Cr-coated Zr-4 claddings are tested in a high-temperature muffle furnace, heated by resistance wires and kept warm for certain times in air. The test temperatures were set at 900 °C, 1000 °C, 1100 °C, 1200 °C and 1300 °C, respectively, with an oxidation time of 1 h. And the lone-term aqueous corrosion tests were conducted in a high-pressure vessel. An accelerated corrosion condition at 420 ± 3 °C, 10.3 ± 0.7 MPa in an aqueous environment for 100 days was set for the Cr-coated and uncoated Zr-4 cladding, simulating the off-normal service condition. Then, the long-term accident resistance of the Cr coating can be evaluated quickly. The deionized water was selected as the corrosion medium in this experiment, avoiding the influence of impurities on the results. And after each stage of the experiment, the deionized water was be replaced. In addition, five specimens were placed in each group of experiments. After each round of testing, a certain number of parallel samples were taken out for structural characterization. The scanning electron microscopy (SEM) equipped with an energy-dispersive spectroscopy (EDS) operating at 20 kV (JSM6700F, JEOL, Toyama, Japan) was utilized to observe the surface and cross-section morphologies of coatings. The electron backscatter diffraction (EBSD) operated at an accelerating voltage of 200 V–30 kV and a scanning step of 0.2 μm (FEI NONA 400, FE-SEM, Thermo Fisher Scientific, Waltham, MA, USA) was used to characterize the microstructure of the coatings. The X-ray diffraction (XRD, D8 Advance, Bruker, Ettlingen, Germany) was applied to analyze the phase constitutions of samples, with a diffraction angle range of 20–80° and a rate of 1°/min. An algorithm describing the sample preparation procedure and plan of experiments is listed in Figure 2 as follows:

## 3. Results

### 3.1. Microstructure of As-Deposited Cr Coatings

**Figure 3:**
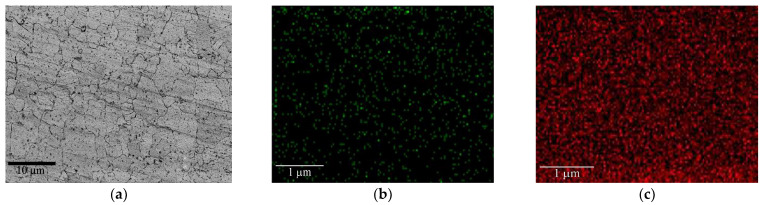
Secondary electronic image of Zr-4 substrate after bombardment by Cr ions (**a**) and the element distribution: (**b**) Cr Mapping, (**c**) Zr Mapping.

Figure 3 shows the secondary electronic morphology and corresponding element mappings of the Zr-4 substrate after the bombardment by Cr ions for 3 min. It can be seen that a lot of dark dots were embedded in the surface of the Zr-4 substrate, and these dark dots were confirmed to be Cr by elemental analysis, indicating the occurrence of Cr ion implantation. This structure is usually intentionally caused by applying a higher negative bias on Cr ions prior to coating deposition, which is good for the growth of the Cr coating subsequently, and also the interfacial bond strength between the substrate and the coating.

**Figure 4:**
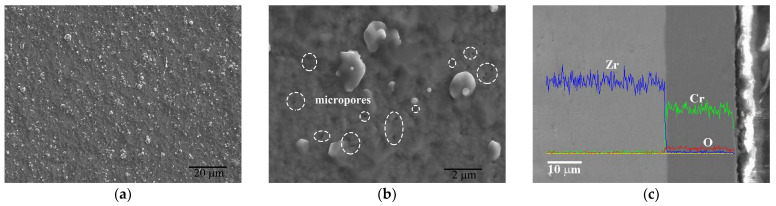
Surface morphology (**a**,**b**) and cross-section view of Cr coating with the EDS line scan (**c**).

The surface and cross-section morphologies of the as-deposited Cr coating are showed in Figure 4. The coating exhibits a typical AIP characteristic morphology with many micron-sized Cr particles distributed on the surface, as shown in Figure 4a. Except for the microparticles, some nanoscale pores as indicated by white dotted circles in Figure 4b (further enlarged) can be clearly observed on the coating surface, which may be attributed to the insufficient diffusion capacity of the absorbed atoms along the surface. These nanopores are highly susceptible to preferential attack by corrosive media, resulting in localized accelerated corrosion. However, no through-hole defect can be seen from the cross-section morphology in Figure 3c. A Cr coating with a thickness of ~19 μm is well adhered to the Zr-4 substrate and exhibits a uniform and relatively compact microstructure.

**Figure 5:**
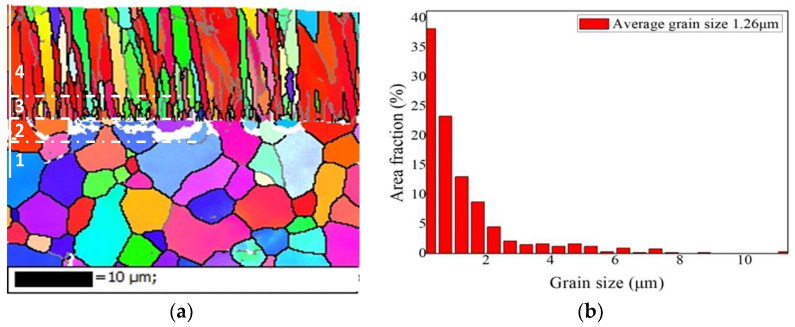
Grain morphology (**a**) and size distribution (**b**) of Cr coating by EBSD.

Figure 5 displays the EBSD pattern of the as-deposited Cr coating and the distribution of the grain size. Four different zones related to the coating growth could be clearly recognized in Figure 5a. Zone No. 1 represents the grain morphologies of the Zr-4 substrate. The grain size of substrate alloy is close to grade 12 based on metallographic grading standards, which is comparable to the uncoated substrate, suggesting that the deposition process of the Cr coating has no obvious effect on the microstructure of the substrate. Zone No. 2 is an interface transition area between the coating and substrate, where a large number of Cr ions or atomic clusters are implanted into the Zr-4 substrate, thus forming a Cr-Zr mixed diffusion interlayer. Obviously, that Cr-Zr mixed zone is formed by the bombardment of Cr ions at a higher bias voltage. This bombardment not only improves the interfacial adhesive strength, but also provides a great quantity of Cr nucleation sites for the growth of subsequent coatings. These Cr nuclei then grow further and form fine equiaxed crystals adjacent to the interface (Zone No. 3). Zone No. 4 is the main part of the Cr coating, exhibiting a columnar crystal structure. As shown in results by XRD, the Cr coating grows in a preferred orientation of (110) and (200). Figure 5b shows the statistical distribution of grain size in the Cr coating. More than 60% of the Cr grains are a size less than 1 μm and the average grain size of the Cr coating is about 1.26 μm. Fine grains will increase the structural compactness of the coating, which is conducive to improving corrosion resistance.

### 3.2. Durability of Cr Coating under Extreme Conditions

**Figure 6:**
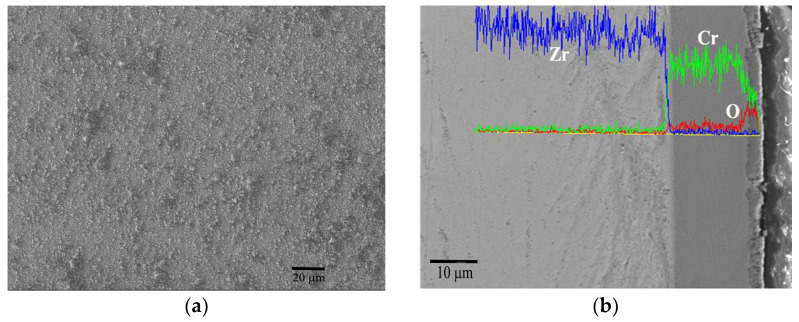
Surface morphology (**a**) and cross-section view (**b**) of Cr coating after oxidation at 900 °C for 1 h with the EDS line scan.

**Figure 7:**
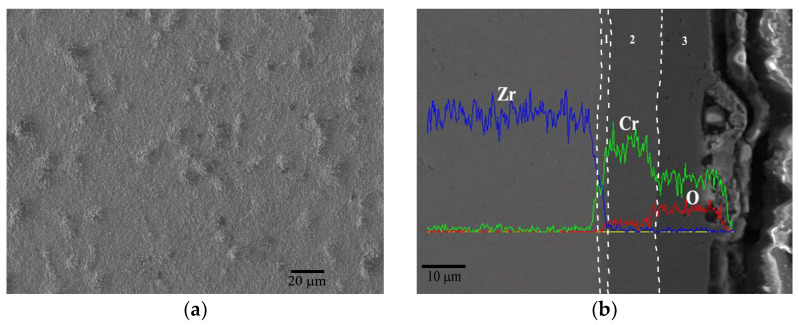
Surface morphology (**a**) and cross-section view (**b**) of Cr coating after oxidation at 1000 °C for 1 h with the EDS line scan.

**Figure 8:**
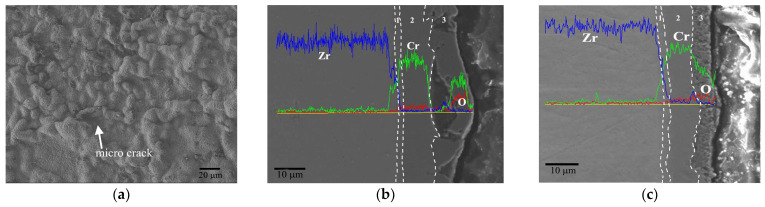
Surface morphology (**a**) and cross-section view (**b**,**c**) of Cr coating after oxidation at 1100 °C for 1 h with the EDS line scan.

**Figure 9:**
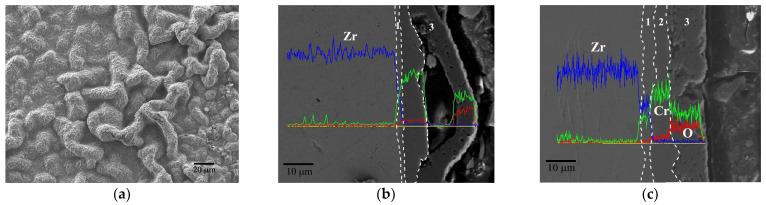
Surface morphology (**a**) and cross-section view (**b**,**c**) of Cr coating after oxidation at 1200 °C for 1 h with the EDS line scan.

**Figure 10:**
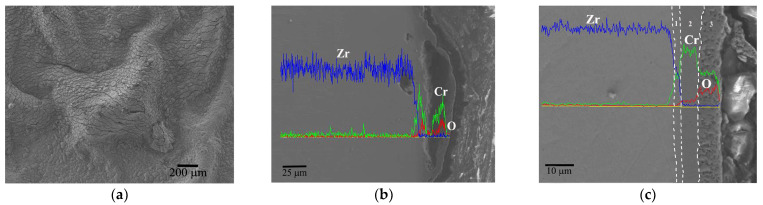
Surface morphology (**a**) and cross-section view (**b**,**c**) of Cr coating after oxidation at 1300 °C for 1 h with the EDS line scan.

Durability performance of Cr coating under extreme conditions can be characterized quickly by isothermal oxidation experiments in air. In order to comprehensively evaluate the resistance against an accidental environment, Cr coated Zr-4 claddings were tested at different elevated temperatures in air. And then the samples were longitudinally cut and polished to observe the cross-sectional morphology of the coating after oxidation. The results are displayed from Figure 6, Figure 7, Figure 8, Figure 9 and Figure 10. Figure 6 shows the surface and cross-sectional morphologies of the Cr coating after oxidation in air at 900 °C for 1 h. Small bulges distributed on the oxidized surface are shown in Figure 6a. and the cross-section view in Figure 6b shows that the outer growing oxide layer is relatively uniform with an average thickness of about 3–4 μm, the residual Cr coating of about 16 μm still adheres well with the substrate. These small bulges are mainly caused by the volume expansion of oxide film. In an oxygen-rich atmosphere, a large number of oxides is generated under the reaction of O and Cr. Also, some O diffuses into the Cr coating through the micro pores and causes local oxidation. Then, the volume expansion of oxides is restricted by the rapid generation of oxides, leading to the formation of local bulges.

When the oxidation temperature rises to 1000 °C, the surface bulges become significantly larger with a size approaching 10 μm (Figure 7a). The cross-sectional image (Figure 7b) reveals that these bulges appear primarily at the outer side of the oxide layer. Except for the bulge defect, the coating can be divided into three parts: the Cr/Zr diffusion zone at the interface (zone No. 1, the same below), the residual Cr coating in the middle (zone No. 2, the same below) and the external Cr_2_O_3_ oxidation zone (zone No. 3, the same below). In the external oxidation zone, there are obvious oxidation pores, which correspond to the bulges in Figure 6a. It is believed that morphology is related to the diffusion corrosion rate in the coating. As the corrosion temperature increases, the diffusion of O into the coating is accelerated, and the corresponding oxidation expansion inside the coating is also intensified. When internal oxides are preferentially generated, the bulging morphologies would be formed in the oxide film by its volume expansion effect, as shown in Figure 7b. It can be predicted that with the further intensification of oxidation, that the bulging morphology inside the coating will gradually increase, and eventually form a macroscopic hole extending to the interface. Although there is no evident spallation of the oxide scale at 1000 °C, its loose structure is undoubtedly unfavorable to the protection of the coating and substrate in more aggressive environments. After oxidation at 1000 °C for 1 h, the thickness of the Cr_2_O_3_ scale increases to ~10 μm and that of the residual Cr coating reduces to about 10, correspondingly.

The surface wormlike bulges further grow and connect with each other when the temperature increases to 1100 °C, as shown in Figure 8a. The cross-section view (Figure 8b) shows a larger hole at the interface between the Cr coating and the oxide layer, which confirms the previous speculation about the expansion of diffusion pores. Some through-wall cracks exist in the oxide layer, which means a more terrible oxidation compared with the results at 1000 °C. The oxide scale is easily spalling off at these defect locations. Figure 8c displays the morphology of the oxide scale at a relatively flat area. The oxide scale exhibits a porous structure with a thickness of around 8 μm while the thickness of the residual coating reduces to about 9 μm. The thinning of the oxide film might be related to its local peeling off during the oxidation process.

When the test temperature further rises to 1200 °C (critical design temperature of the nuclear reactor), as seen in Figure 9a, the wormlike bulges expand significantly, forming larger bubbles with tens of microns in size. The holes in the cross-sectional image also extend along the interface and become larger (Figure 9b). Similarly, in the area without the bulge and interface crack, as shown in Figure 9c, the oxide film is loose and porous with a thickness of about 9 μm, but the residual Cr coating of about 4 μm strongly adheres to the underlying substrate.

At 1300 °C, the oxidation of the Cr coating is further aggravated, as shown in Figure 10. The size of the bulges rapidly increases to hundreds of microns, and full of micro-cracks on the surface (Figure 10a). That morphology is mainly caused by the excessive volume expansion of the oxide film at an extremely high temperature. The cross-sectional morphology in Figure 10b shows that the Cr coating in the bulge area has been consumed totally, and the oxide scale may suffer severe cracking and spalling, leaving only an extremely thin discontinuous oxide layer. In the non-bulge area, as shown in Figure 10c, the penetrating fracture of the surface oxide layer is clearly visible, and the residual Cr coating is less than 4 μm. The oxidation bulge on the surface accelerates the corrosion deterioration of the Cr. However, it can still be believed that the Cr coating has the ability to protect the Zr-4 substrate at 1300 °C for 1 h at least.

**Figure 11:**
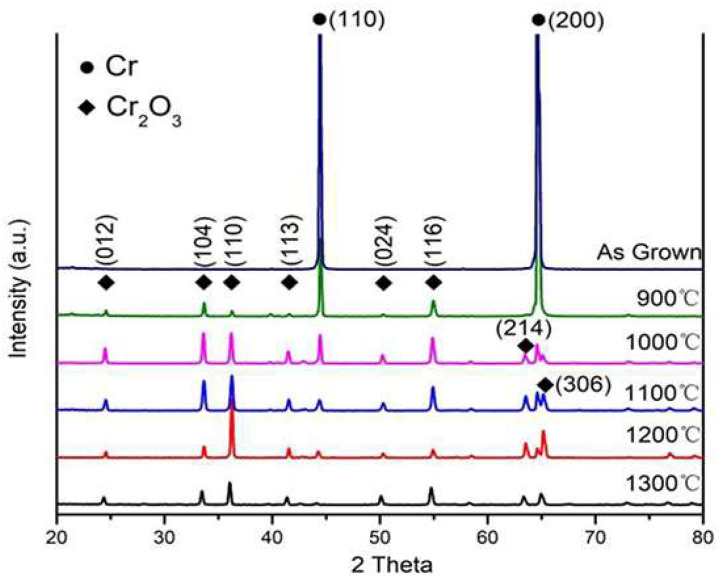
XRD of Cr coatings at various temperatures.

Figure 11 shows the XRD patterns of the Cr-coated Zr alloys after oxidation at 900–1300 °C. The as-deposited Cr coating shows strong diffraction peaks of (200) and (110). After oxidation at 900 °C for 1 h, weak Cr_2_O_3_ gradually strengthens while those of the Cr coating gradually weaken and finally disappear after oxidation at 1300 °C, implying that the oxidation of the Cr coating is aggravated at higher temperatures. However, it should be noted that no Zr or Zr oxides were detected by the XRD, suggesting that the Zr-4 substrate is protected by the Cr coating well.

### 3.3. Durability of the Cr Coating under Extreme Conditions

#### 3.3.1. Evolution Behavior of the Cr Coating

**Figure 12:**
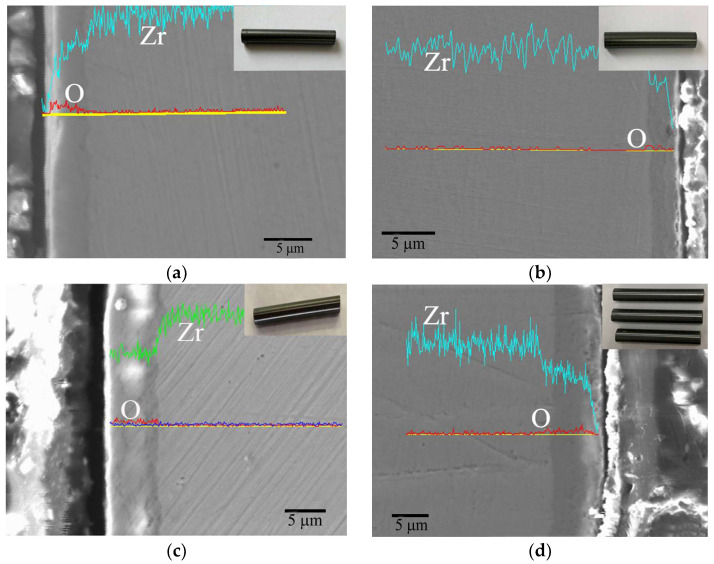
Cross section view with EDS line scan of uncoated Zr-4 alloy in a long-term aqueous test at 420 ± 3 °C, 10.3 ± 0.7 MPa for (**a**) 25 days, (**b**) 50 days, (**c**) 75 days and (**d**) 100 days.

After 14 days of corrosion, there was no change in the appearance of the Cr-coated Zr-4 cladding, presenting a shiny metallic color the same as deposited, while a black oxide film was formed on the surface of the Zr-4 cladding tube. After recording the weight gain rate of the samples, there is no need for further analysis. The cross-sectional morphologies and corresponding EDS line scans of the Zr-4 cladding after corrosion for 25–100 days are shown in Figure 12. The surface of the Zr-4 cladding was covered by the black oxide products as shown in the insert optical images, indicating that severe corrosion has occurred. After corrosion for 25 days, the thickness of the oxide scale grown on the Zr-4 substrate is 4 μm (Figure 12a), and gradually increases to about 5 μm at 50 days (Figure 12b), 6 μm at 75 days (Figure 12c) and 9 μm at 100 days (Figure 12d). As corrosion progresses, the growth rate of the oxide film slows down compared to that of the initial period, suggesting that the formation of a dense oxide layer has a certain protective effect on the Zr-4 substrate. However, after 75 and 100 days of corrosion, the out layer of the oxide film became loose as shown in Figure 10c and Figure 12d, which weakens its protection and leads to a slight increase in its growth rate.

**Figure 13:**
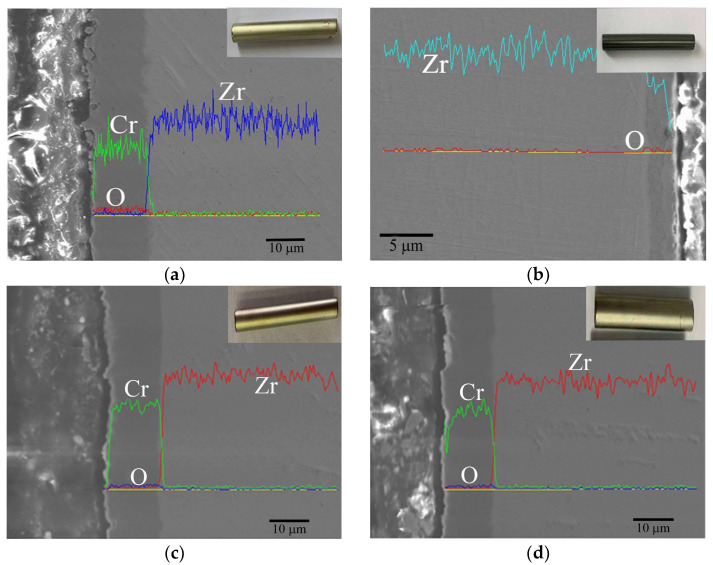
Cross section view with EDS line scan of Cr-coated Zr-4 alloy in a long-term aqueous test at 420 ± 3 °C, 10.3 ± 0.7 MPa for (**a**) 25 days, (**b**) 50 days, (**c**) 75 days and (**d**) 100 days.

In contrast, the Cr-coated Zr-4 cladding displays excellent corrosion resistance in the same service environment as shown in Figure 13. Macroscopically, all the Cr-coated Zr-4 samples remain intact after corrosion, without bulging or peeling. Microscopically, Cr coating exhibits different corrosion characteristics. After 25 days of corrosion, there is no obvious change in the coating thickness, whereas minor indications of cracking and shedding are observed in the specific areas on the outer surface of the Cr coating (Figure 13a). As the corrosion times extend to 50 days, minor cracks or fractures can be seen to exist in Figure 13b, and the diffusion of oxygen is also inhibited. The thickness of the Cr coating is reduced to about 15 μm. The same phenomenon also occurs at the corrosion cycle of 75 (Figure 13c) and 100 (Figure 13d) days. Due to the corrosion, an uneven morphology is exhibited on the coating surface. The residual Cr coating is about 12 μm and 9 μm. It can be believed that the Cr coating shows a gradual thinning corrosion mechanism under the long-term aqueous test.

**Figure 14:**
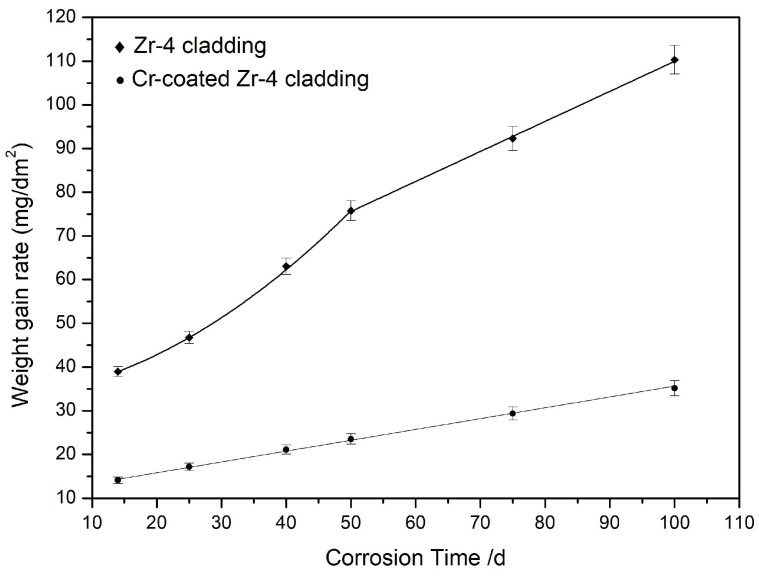
Weight gain rate of Zr-4 and Cr-coated Zr-4 cladding in a long-term aqueous test at 420 ± 3 °C, 10.3 ± 0.7 MPa.

The corrosion phenomenon of the Cr coating can also be reflected from its corrosion kinetics curve. Figure 14 shows the weight gain rate of uncoated and Cr-coated Zr-4 cladding after corrosion at 420 ± 3 °C, 10.3 ± 0.7 MPa for 100 days. It should be noted that in this test, the Zr-4 claddings were only coated by Cr on the outer surface, while none was in the inner. The corrosion kinetics curve of the Zirconium alloys can be divided into two stages from Figure 13: slow corrosion in the initial stage and accelerated corrosion in the subsequent stage. The growth kinetics curve of the oxide film in the initial stage follows a parabolic law before 50 days of corrosion. Afterwards, the corrosion is accelerated, following a linear kinetic law from 50 days to 100 days. N. Ni and Cox B. also have observed similar experimental results and have performed further research [34,35]. They believed that the structural transformation of Zirconium oxides, from a tetragonal phase to a monoclinic phase during corrosion, is the reason for the accelerating corrosion rate. In this study, the corrosion rate inflection points of the Zr-4 cladding occur around 50 days after corrosion. However, the corrosion kinetics curve of the Cr-coated Zr-4 cladding is more in line with a simple linear law, which means a uniform corrosion mode. The average corrosion gain rate of the Cr coating is only about 1/3 of the uncoated Zr-4 cladding, which is a lower than normal value, 1/2, based on existing studies [36]. The decrease in the corrosion weight gain rate precisely confirms the theoretical analysis of the corrosion mechanism regarding the gradual thinning of the Cr coating.

#### 3.3.2. Inhibition of Hydrides by Cr Coating

Hydrides can seriously affect the mechanical properties of the Zirconium alloy. Microcracks often are caused by hydrides, ultimately leading to the brittle fracture of the Zr-4 cladding. Therefore, characterizing the hydrides and their orientation in the service environment is very important for evaluating the mechanism effect of the Cr coating on the Zr-4 cladding. In this study, after corrosion in a long-term aqueous test, the longitudinal section metallographic of the Zr/Cr samples was prepared and immersed into a mixed acid solution of 1% HF, 45% HNO_3_ and 45% H_2_O_2_ lasting about 35 s. Then, the metallographic microscope was used to observe the hydrides. Figure 14 shows the hydride distribution metallographic maps of the uncoated and Cr-coated Zr-4 substrates after corrosion at 420 ± 3 °C, 10.3 ± 0.7 MPa for 50 days and 100 days, respectively.

**Figure 15:**
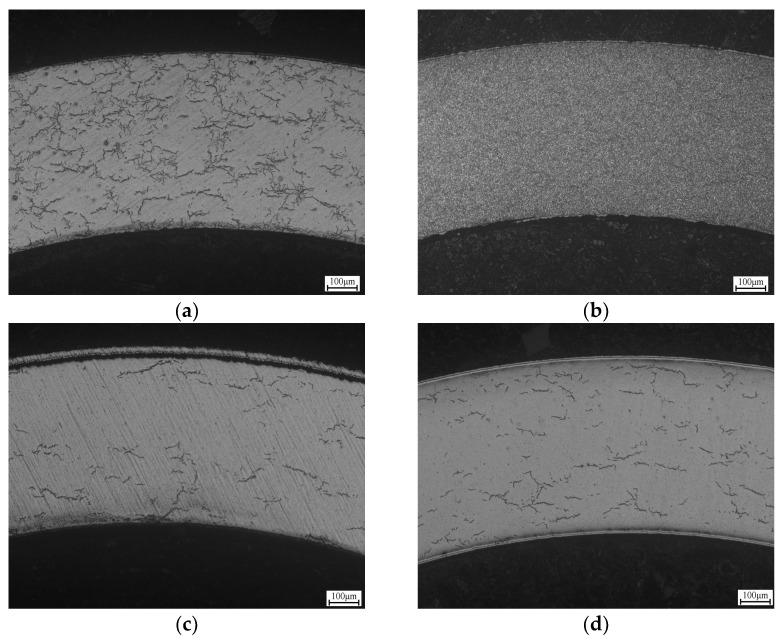
Hydride distribution metallographic maps of uncoated (**a**,**b**) and Cr-coated Zr-4 substrates (**c**,**d**) after corrosion at 420 ± 3 °C, 10.3 ± 0.7 MPa for 50 days (**a**,**c**) and 100 days (**b**,**d**).

After 50 days of corrosion, large amounts of hydrides are precipitated inside the Zr-4 cladding and interwoven with each other, forming the complex hydride texture in Figure 15a. This structure is more conducive to the all-direction rapid initiation and propagation of microcracks in the substrate. The Zr-4 cladding has been filled with hydrides and it is impossible to distinguish the orientation after 100 days, as shown in Figure 15b, which means a highly brittle fracture risk for the Zr-4 cladding.

But the Cr-coated Zr-4 substrate shows a different situation after corrosion, with a significant inhibition of hydrides. The number of hydrides is reduced by nearly 90% compared to the uncoated substrate after 50 days of corrosion, as shown in Figure 15c. After 100 days of corrosion, the number of hydrides in the substrate has hardly increased, as shown in Figure 15d. Even if the axial hydrides are not observed, it can be concluded that the precipitation of a hydride is significantly restrained by the Cr coating.

Due to the relatively high chemical inertness of the Cr coating and also the extremely low affinity between Cr and H, the Cr coating acts as a passivation layer for the Zr-4 cladding in high-temperature water environments and inhibits the diffusion of the H element. The hydrogen produced in the corrosion process is difficult to penetrate into the Zr-4 substrate. As a result, the precipitation of natural hydride decreases. So, it can be believed that the Cr coating can significantly reduce the brittle fracture risk of the Zr-4 alloy cladding tubes under a long-term service condition.

## 4. Discussion

### 4.1. Oxidation Mechanism of Cr Coating in Hot Air

According to the evolutionary trends of cross-sectional morphologies of Cr coating at different oxidation temperatures, it can be found that the generated oxide film is not as dense as expected, with many tiny holes. The bulging and cracking along the interface worsen with increasing temperature. As we all know, the deposition of the Cr coating is a process of particle accumulation, forming a morphology as shown in Figure 3a. Inevitably, there are many fine gaps or holes between the particles. During the corrosion process, besides the oxidation reaction at the surface, some O would diffuse into the Cr coating through these gaps or holes, causing oxidation inside the coating. The expansion of the internal oxide volume causes its structure to become loose. As the temperature increases, the rate of oxide generation accelerates and the volume expansion intensifies, leading to more voids at the interface between the oxide film and the residual Cr coating. These voids will accumulate with each other to form holes, which will eventually become the direct inducement of the cracking of the Cr_2_O_3_ film and the continuous corrosion of the residual Cr coating.

Obviously, the bulging and cracking occurring at the interface between the residual Cr coating and the oxide film is caused by the release of internal stress. Researchers have conducted a theoretical study of the stress during the oxidation process of coatings. The results show that the growth stress of the Cr_2_O_3_ film during oxidation is compressive stress, and the value can be calculated by the following formula [23,37]:

σ = −2E0(m − 1)/[3(1 − υ0)m] · (h/R)(1)

where σ is the stress (GPa), E0 is Young’s modulus, υ0 is Poisson’s ratio, h is the oxide layer thickness, R is the interfacial curvature radius, m is the ratio of the newly generated oxides to the consumed metals, its value is about 1.3 at 1200 °C. Assuming that h/R is approximately equal to 0.1, the value of E0 and υ0 are 178 GPa and 0.3, respectively. It can be calculated that the internal stress of Cr_2_O_3_ at 1200 °C is about 3.91 GPa. As the oxidation process continues, the thickness of the Cr_2_O_3_ film increases further, the value of h/R increases synchronously, resulting in a further increase in internal stress. The higher stress can be released through the creep of the oxide film and finally lead to the bending of the oxide layer on the surface. And the oxide film completely loses its protective effect on the coating.

**Figure 16:**
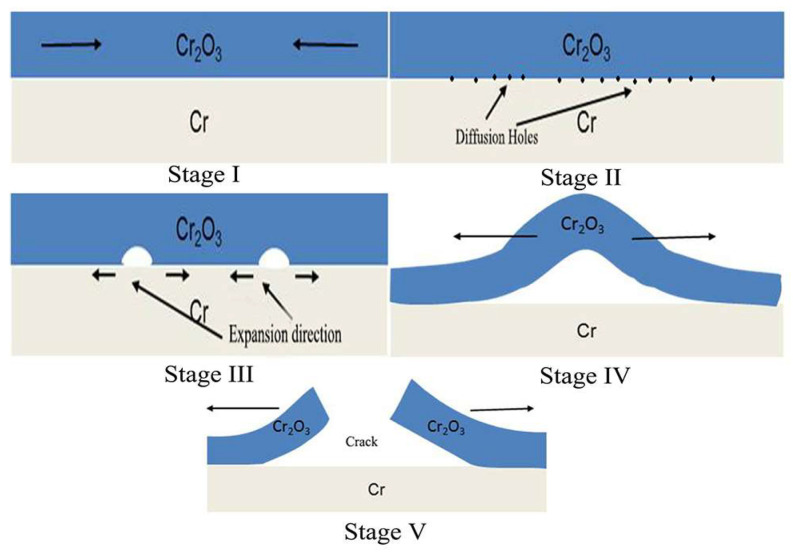
Corrosion deterioration mechanism of the Cr coating.

Figure 16 shows the dynamic mechanism of fracture along the interface of the Cr_2_O_3_ film and the residual Cr coating. At the initial stage of oxidation, a dense and uniform oxide film is rapidly formed; the internal stress along the direction shown in Stage I is generated simultaneously, as shown in Figure 6. As the temperature increases and the corrosion time prolongs, more voids at the interface are formed due to the acceleration of the generation and the volume expansion of the oxide (Stage II), forming interface holes by connecting with each other. From Figure 7, the hole in the Cr_2_O_3_ film and the interface of the Cr/Cr_2_O_3_ perfectly confirms this viewpoint. Internal stress is further applied on these holes, causing them to expand along the interface to create larger defects, as shown in Stage III, which can be seen in Figure 7 and Figure 8. When the internal stress in the Cr_2_O_3_ film accumulates to a certain extent, creep occurs, resulting in the creation of worm-like bulges, as shown in Figure 8a and Figure 9a. And these worm-like bulges preferentially form and expand at the interface holes, as shown in Stage IV (Figure 8b and Figure 9b). When the bulge reaches the deformation limit, it ruptures and falls off, as shown in Stage V (Figure 10). The residual Cr coating is exposed to the corrosive medium and the corrosion process continues until it is consumed totally. So, it can be believed that the failure of the Cr coating under a high temperature can be attributed to the continuous formation, bulging, cracking and peeling of the loose Cr_2_O_3_ film on the surface.

### 4.2. Corrosion Mechanism of Cr Coating in Long-Term Aqueous Test

For the uncoated Zr-4 substrate, the black oxide film generated on the surface will gradually thicken as the corrosion time prolongs in long-term aqueous test. Unfortunately, the density of the oxide film also decreases. The loose structure of the oxide scale would act as a diffusion channel for the corrosive medium, resulting in the severe corrosion of the Zr-4 substrate. It can be predicted that when the corrosion time is extended, the oxide film will further thicken and be highly likely to fall off, deteriorating the mechanical properties and thermal conductivity of the Zr-4 cladding tube and leading to catastrophic failure.

However, the Cr coating shows a good protective effect on the Zr-4 substrate in the long-term aqueous test, hindering the corrosion reaction, suppressing the precipitation of hydrides, and delaying the time cycle of the hydrogen-induced cracking of the Zr-4 substrate. However, with the prolonging of the corrosion time, the coating showed a significant thinning phenomenon. Then, the question is how the Cr coating disappears.

Answers can be found from the experiment phenomena and corrosion theory. In theory, Cr coatings will spontaneously generate a dense oxide film in corrosive media. However, no significant formation of an oxide film was found in this experiment, only a little amount of O present in the coating proved by the line scan of EDS, which means an ultra-thin oxide film on the surface. Brachet et al. also have confirmed the existence of that nanoscale oxide films on the surface [2]. Moreover, the peeling and cracking of the Cr coating can be seen clearly and the thickness of the Cr coating also decreased. Obviously, the oxidation of Cr under a high partial pressure of water vapor at a high temperature showed different behavior than that under dry oxygen or dry air with respect to the surface morphology, microstructure and adhesion of the oxide scale, and volatile oxyhydroxide formation [29,38,39]. Oxides are likely to dissolve under high-pressure water vapor, or fall off in a loose state. Based on the analysis, potential reactions for the formation of Cr oxide and volatilization under pure steam conditions are summarized below [40].

Cr(s) + 3H_2_O(g) = Cr_2_O_3_(s) + 3H_2_(g) (2)

1/2Cr_2_O_3_(s) + H_2_O(g) + 3/4O_2_(g) = CrO_2_(OH)_2_(g) (3)

2Cr_2_O_3_(s) + H_2_O(g) = 2CrO_2_(OH)2(g) + 1/2O_2_(g) (4)

**Figure 17:**
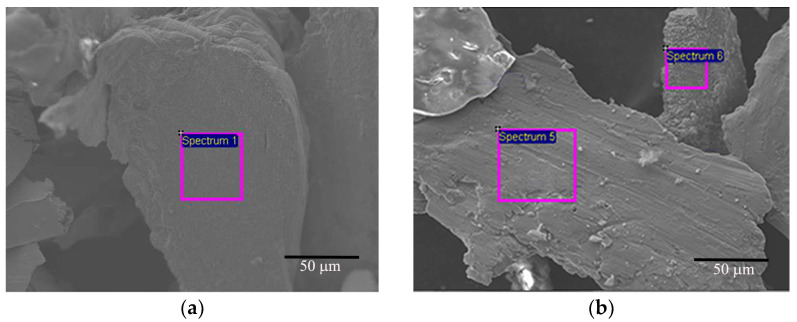
Corrosion residues (**a**,**b**) with EDS after a long-term aqueous test at 420 ± 3 °C, 10.3 ± 0.7 MPa for 100 days.

According to these reaction formulas, the volatile chromium oxyhydroxide is formed by the chemical reaction between the Cr oxides and water under the corrosion condition of 420 ± 3 °C, 10.3 ± 0.7 MPa. Therefore, it can be considered that the formation of volatile chromium oxyhydroxide is one of the reasons for the loss of oxides. Opila et al. [41] also have predicted that this phenomena would become a prominent phenomenon if water vapor partial pressure was higher than 10 kPa. On the other hand, some corroded residues were found in the water, and an EDS analysis (Figure 17) was performed and the results were listed in Table 2. As it can be seen, most of these residues were exfoliated Cr coatings or Cr oxides. Therefore, the detachment of oxides and some Cr coatings is also the cause of coating thinning.

Based on the above experimental phenomena and theoretical analysis, the corrosion mechanism of the Cr coating under the corrosion condition of 420 ± 3 °C, 10.3 ± 0.7 MPa can be illustrated in Figure 16.

**Figure 18:**
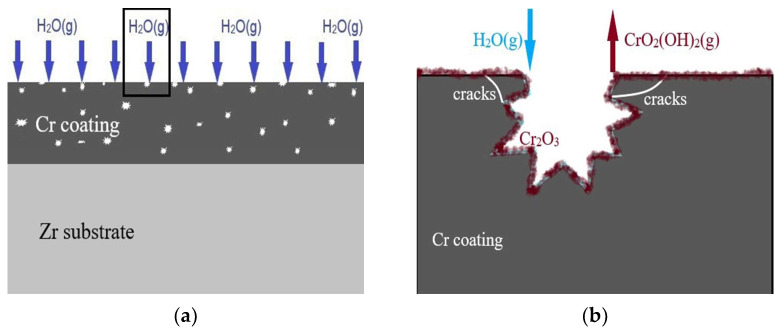
Corrosion mode of PVD-Cr-coated Zr-4 alloy in a long-term aqueous test at 420 ± 3 °C, 10.3 ± 0.7 MPa. (**a**) initial state of corrosion, (**b**) entire corrosion process.

The entire corrosion process can be described as follows: Some nanoscale pores exist in the Cr coating due to the island nucleation and columnar crystal growth mode of physical-vapor-deposition technology, as shown in Figure 4b and Figure 18a. Then, the water would penetrate into the coating through these nanoscale pores under high pressure and react with the surrounding Cr at a high temperature, resulting in the generation of the Cr oxidation. The expansion of the oxide volume inside the micro pore will promote the formation of microcracks, leading to the delamination of the Cr coating. Also, the volatile chromium oxyhydroxide, CrO_2_(OH)_2_, is formed, leading to the dissolution and volatilization of oxides. The synchronous occurrence of the above corrosion process constitutes the behavior of the Cr coating in a long-term aqueous test. Figure 18b illustrates the entire corrosion process of the Cr-coated Zr-4 cladding tube. The cycle of the detachment and hydrolysis reaction will cause the continuous thinning of the Cr coating.

In addition, the service life of Cr-coated Zr-4 cladding can be predicted based on corrosion kinetic curves. From Figure 14, the corrosion process of the Cr-coated Zr-4 cladding at 420 ± 3 °C, 10.3 ± 0.7 MPa conforms to a linear law. The generation, delamination and chemical reaction in water of oxides are dynamic and stable processes, and also the corrosion rate remains stable. According to the dynamic curve trend in Figure 14, it takes at least 200 days for the Cr coating to be completely consumed. So, it can be predicted in this study that the service life of the Cr-coated Zr-4 cladding can be extended by an additional 200 days at least.

### 4.3. Perspectives about Cr Coating for ATF

The research results demonstrate that the Cr coating has a significant protective effect on the Zr-4 substrate under normal and off-normal conditions. It can be expected that the common application problems, such as hydride precipitation and nodular corrosion, could be completely avoided by the deposition of the Cr coating on the fully enclosed Zr-4 cladding tubes, and the refueling cycle of the nuclear reactor will be extended by at least 200 days.

However, the analysis of the corrosion degradation mechanism of the Cr coating also suggests that the deposition defects in the coating are one of the main reasons for coating failure. The oxide film generated on the PVD-Cr coating in corrosive environments is not as dense as expected, mainly caused by the deposition defects. In addition, the dissolution reaction of oxides in water also causes the continuous corrosion of the Cr coating. Assuming that the dissolution reaction is inevitable, studying how to improve the consistency of the coating structure and eliminate deposition defects remains the research topic for accident resistant Cr coatings. What is more, for the engineering implication of the Cr coating, more attention should be paid to the uniform deposition of the coating and stability of the preparation process on the full-size cladding tube, for the purpose of controlling the uniform corrosion of the Cr coating.

In addition, due to the special service environment of the fuel cladding, the corrosion damage and failure of materials under neutron irradiation, such as DPA (displacement per atom) corrosion, is the primary key issue to consider. It is also necessary to consider whether the corresponding corrosion problem will occur in Cr coatings under neutron irradiation. Some new theoretical calculation methods can better help us analyze the damage mechanism of Cr coating shells under neutron irradiation conditions [42,43].

## 5. Conclusions

The Cr coatings with a thickness of about 19 μm were deposited on Zr-4 cladding, and a typical columnar crystal structure was observed by SEM and EBSD. Micropores left in the coating due to the particle stacking deposition mode will have a negative effect on the corrosion resistance of the PVD-Cr coating.

The isothermal oxidation experiment results showed that the loose and porous oxide film is formed and gradually shows a (110) crystal-preferred orientation. As there is an increase in temperature in the air, the Cr_2_O_3_ is gradually separated from the interface due to voids at the interface and the increase of internal oxidative stress. Obviously, the cyclic corrosion mechanism of void initiation–propagation–cracking at the oxide film interface is the root cause of the Cr coating’s corrosion deterioration in air. In the long-term aqueous test at 420 ± 3 °C, 10.3 ± 0.7 MPa for 100 days, the Cr coating exhibits a uniform thinning corrosion mechanism. It is believed that the thinning of the Cr coating is related to the detachment of the oxide film and local Cr coating, as well as the dissolution of the partial oxide film. But the Cr coating has a significant inhibitory effect on the generation of internal hydrides if it is not consumed totally.

More efforts should be made to optimize the PVD-Cr coatings. The study of the structural density of the coating, the uniform deposition of the coating and the stability of the preparation process on the full-size cladding tube are equally important. Detailed research is also needed on the corrosion mechanism of Cr coatings under engineering service conditions, especially under neutron irradiation.

## Figures and Tables

**Table 1:** Basic chemical composition of Zr-4 alloy.

Zr	Sn	Fe	Cr	Ni	Si	Hf
In balance	~1.5	~0.2	~0.1	~0.12	~0.9	~0.04

**Table 2:** EDS results of the corrosion residues.

	C	O	Cr	Fe
Spectrum 1	/	3.85	95.23	0.92
Spectrum 5	1.03	48.80	50.17	/
Spectrum 6	/	47.36	51.32	1.32

## Data Availability

Data are contained within the article.
